# A Family With Factor VII Deficiency: A Possible Autosomal Dominant Transmission

**DOI:** 10.7759/cureus.82526

**Published:** 2025-04-18

**Authors:** Zheng Song, Stephenie Garcia, Thiagarajan Nandhagopal, Stanley Kim

**Affiliations:** 1 Family Medicine, Rio Bravo Family Medicine Residency Program, Bakersfield, USA; 2 Pediatrics, Kern Medical Center, Bakersfield, USA; 3 Hematology and Oncology, Kern Medical Center, Bakersfield, USA

**Keywords:** autosomal dominant inheritance, coagulation disorder, factor vii deficiency, genetic transmission, hereditary bleeding disorder

## Abstract

Coagulation factor VII plays a crucial role in the extrinsic pathway of the coagulation cascade. Deficiency of coagulation factors, including factor VII, is a rare but recognized cause of abnormal uterine bleeding (AUB) in women. This case report presents an 11-year-old previously healthy girl who arrived at the emergency department with 18 days of heavy menstrual bleeding and hemodynamic instability due to severe anemia. Laboratory evaluation revealed prolonged prothrombin time (PT/INR) with a normal partial thromboplastin time (PTT). A factor VII activity assay confirmed a severely decreased serum factor VII level. Bleeding was successfully managed with recombinant human factor VII. Further family screening showed reduced factor VII activity in the patient’s mother and three siblings, while the father’s levels were normal, suggesting a possible dominant inheritance pattern. However, genetic testing is required to confirm this hypothesis.

## Introduction

Factor VII deficiency, previously known as serum prothrombin conversion accelerator (SPCA), was first identified by B. Alexander and colleagues in 1951. The discovery stemmed from the case of a four-year-old girl admitted to the Children's Medical Center in Boston (now Boston Children's Hospital) with a three-day history of hematemesis and bloody stools. Laboratory tests revealed a markedly prolonged prothrombin time of 72 seconds [1].

Coagulation factor VII plays a crucial role in the extrinsic pathway of the coagulation cascade and also contributes to the intrinsic pathway by activating factor IX [2-5]. The clinical manifestations of factor VII deficiency vary widely, ranging from mild symptoms, such as epistaxis, gum bleeding, and easy bruising, to more severe complications, including postoperative bleeding, hematomas, hematuria, and hemarthrosis. In rare cases, life-threatening events, such as spontaneous intracranial or gastrointestinal hemorrhage, may occur [2,6]. Notably, the clinical phenotype does not always correlate with genotype or serum factor VII activity. Bleeding symptoms have been observed in both homozygous and heterozygous individuals. One study reported symptoms in 71% of homozygous, 50% of compound heterozygous, and 19% of heterozygous individuals. While some homozygous individuals may remain asymptomatic, severe hemorrhages, such as intracranial or gastrointestinal bleeding, are uncommon in heterozygous patients. In women, Factor VII deficiency can also present with severe menorrhagia [4-7].

Unlike factor VIII and IX deficiencies, which follow an X-linked inheritance pattern, factor VII deficiency is inherited in an autosomal recessive manner [3]. Early studies of affected families demonstrated that individuals with the disorder typically have heterozygous (carrier) parents who remain asymptomatic [1,8]. This inheritance pattern was further confirmed through genetic analyses of affected individuals [9-11]. The rarity of severe factor VII deficiency - estimated at one in 500,000 - aligns with the expected frequency of autosomal recessive disorders. However, some heterozygous individuals exhibit significantly reduced factor VII activity and may experience severe bleeding episodes, such as hematuria or hemarthrosis [6]. This raises the possibility of a dominant-negative effect, similar to that observed in factor XI deficiency [12,13].

We present a family affected by factor VII deficiency, in which both daughters experienced prolonged bleeding episodes, leading to severe anemia, necessitating blood transfusions and recombinant factor VII treatment. Blood tests revealed varying degrees of reduced factor VII activity in the mother and all three children, while the father remained unaffected with normal factor VII levels.

## Case presentation

In April 2024, an 11-year-old girl presented to the hospital with her father due to 18 days of heavy menstrual bleeding and dizziness. According to the patient, this was her second menstrual cycle. Her menarche in February 2024 lasted only three days. However, her current episode had persisted for 18 days without improvement, with bleeding severe enough to soak through a pad every two hours. She was not taking any prescribed or over-the-counter medications, supplements, or herbal remedies. Aside from dizziness, she denied experiencing chest pain, shortness of breath, or gastrointestinal or genitourinary symptoms.

According to the patient and her father, there is no known family history of excessive bleeding, except for the patient’s nine-year-old sister. A few years prior, the sister experienced prolonged bleeding for four days following a tooth extraction, leading to severe anemia with a hemoglobin level of 3.3 g/dL. She was diagnosed with factor VII deficiency and treated with a blood transfusion and recombinant factor VIIa at Valley Children's Hospital. Since then, she has had no further bleeding episodes and continues to be monitored annually by a hematologist. Other immediate family members (both parents and the patient's brother) reported no personal history of abnormal bleeding or easy bruising. The mother specifically denied heavy menstrual bleeding, postpartum hemorrhage, or need for transfusion during deliveries. Most interestingly, the patient herself had no prior bleeding manifestations before her acute presentation, despite subsequently confirmed severe factor VII deficiency (<1%).

Upon arrival at the emergency department, the patient's initial vital signs were within normal limits, except for an elevated heart rate of 120 bpm. Laboratory tests (Table 1) revealed severe anemia with a hemoglobin level of 5.8 g/dL. She received two units of blood transfusion; however, bleeding persisted despite treatment with oral medroxyprogesterone acetate, intravenous conjugated estrogen, and intravenous aminocaproic acid. Given the patient’s family history of factor VII deficiency in her sister, a hematologist from Valley Children's Hospital recommended treatment with recombinant human factor VII. The patient received a total of four intravenous doses of recombinant factor VII (1 mg each). Following administration, her bleeding significantly decreased to less than 10 mL over 12 hours and eventually stopped completely. She maintained a stable hemoglobin level of 8 g/dL and was discharged home with oral birth control pills and iron supplementation. Coagulation studies later confirmed a factor VII activity level of <1% (reference range: 60-150%), indicating severe factor VII deficiency.

**Table 1:** Laboratory findings RBC: red blood cells; Hbg: hemoglobin; MCV: mean corpuscular volume; WBC: white blood cells; HCT: hematocrit; MCHC: mean corpuscular hemoglobin concentration; RDW: red cell distribution width; APTT: activated partial thromboplastin time; mm³: cubic millimeter; gm/dL: grams per deciliter

Laboratory Findings	Values	Normal Values
Urine HCG	Negative	___
Blood Type	A positive	___
Antibody screen	Negative	Negative
Total WBC count	4700/mm^3^	5000-14,000/mm^3^
Total RBC count	2.65 million cells per microliter (cells/mcL)	3.98-5.29 million cells per microliter (cells/mcL)
Hgb	5.8 gm/dL	11.5-14.7 gm/dL
MCV	68.6 femtoliters	75-90.6 femtoliters
HCT	18.2%	33.9-43.3%
MCHC	31.9 gm/dL	22.4-36.0 gm/dL
RDW	15.5%	11.3-13.9%
Monocytes%	7.6%	2.0-12.0%
Lymphocytes%	24.5%	29.0-44.0%
Eosinophils%	0.0%	<=6.0%
Basophils%	0.7%	<=2.0%
APTT	25.9 seconds	24.5-35.0 seconds
Prothrombin time	37.5 seconds	12.1-14.2 seconds
INR	4	<1.1

**Figure 1:**
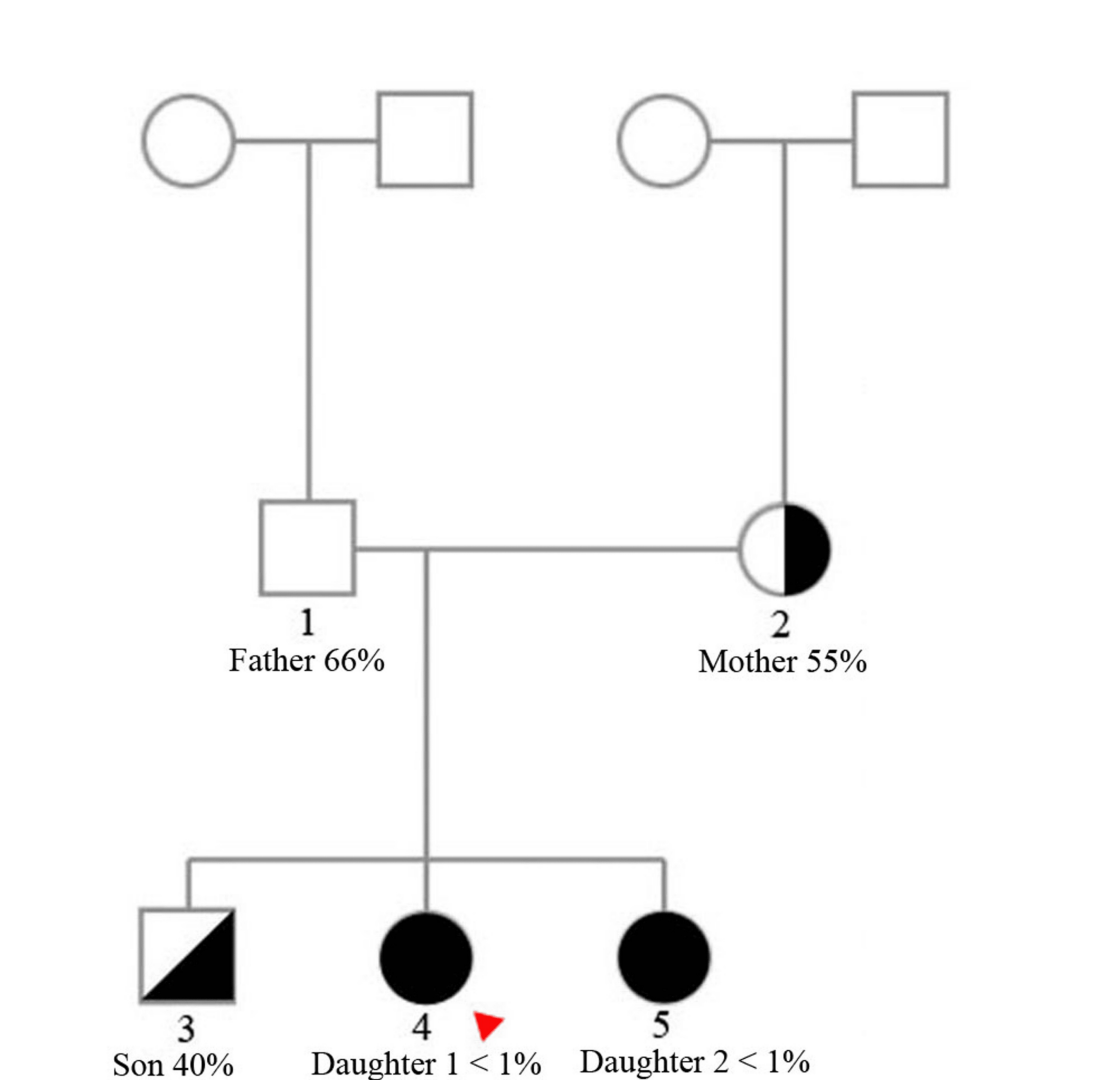
Patient family pedigree with each member's factor VII activity Red arrow: patient

Interestingly, further blood tests revealed a heterogeneous pattern among family members (Figure 1). The mother and all three children exhibited varying degrees of reduced factor VII activity, while the father remained unaffected with normal factor VII levels (Table 2). This family, however, declined further genetic testing.

**Table 2:** Lab results among family members (Daughter 1 is the patient) PT: prothrombin time; INR: international normalized ratio; PTT: partial thromboplastin time, activated

	PT (9.0-11.5 Sec)	INR (0.9-1.1)	aPTT (23.0-32.0 sec)	FVII activity (60-100%)
1. Father	11.9	1.1	28	66%
2. Mother	11.6	1.1	27	55%
3. Son	13	1.2	27	40%
4. Daughter 1	32.7	3.2	24.3	<1
5. Daughter 2	38.5	3.8	27	<1

## Discussion

Based on our literature review of PubMed in October 2024, our case report is the only one on pediatric patients with severe factor VII deficiency presenting with menorrhagia that led to life-threatening severe bleeding. Additionally, our patient did not have any history of easy bleeding prior to the start of her menstrual period. The only clue that led to her diagnosis is her younger sister’s history of factor VII deficiency. Previous articles on this topic were primarily based on retrospective chart reviews [14,15]. The combination of severe factor VII deficiency (<1%) in our patient and her lack of previous bleeding history demonstrates again the importance of considering bleeding disorders in adolescents presenting with abnormal uterine bleeding (AUB) [14-19]. Even though factor VII deficiency is a rare cause of AUB among adolescents, one hemophilia treatment center in Florida found factor VII deficiency in about 20% of adolescent females referred to their center for heavy menstrual bleeding (HMB) and iron deficiency anemia (IDA) [15].

Factor VII activity may be decreased in the setting of vitamin K deficiency or liver disease. However, our patient has no known history of liver dysfunction. In the United States, vitamin K deficiency is exceptionally rare in both children and adults. When present, it is most often associated with conditions such as severe malnutrition, prolonged use of broad-spectrum antibiotics, dependence on total parenteral nutrition, or underlying malabsorption syndromes - none of which are applicable to our patient [4].

Factor VII deficiency is typically inherited in an autosomal recessive manner [2,11]. This means that, when both parents are heterozygous carriers, there is a 25% chance that offspring will inherit two mutated alleles and be affected, a 50% chance they will be asymptomatic carriers, and a 25% chance they will inherit two normal alleles and remain unaffected [20,21]. In this family, the mother and all three children exhibited reduced factor VII activity. Both daughters had severely decreased factor VII levels and a history of prolonged bleeding, while the son had a milder reduction in factor VII activity without symptoms. The father had normal factor VII levels. One possible explanation is compound heterozygosity, where each parent carries a different mutation in the *F7* gene, of which over 200 mutations have been identified [22]. In this scenario, the mother’s mutation may be more severe, leading to significantly decreased factor VII levels, while the father’s mutation may be milder, allowing for normal factor VII production. The children could have inherited either one (as in the son) or both (as in the daughters) of these mutations, resulting in varying phenotypic presentations between the affected daughters and the asymptomatic son.

An interesting alternative explanation for this family's phenotypic presentation is the possibility of an autosomal dominant inheritance pattern, similar to Hemophilia C (factor XI deficiency), which can exhibit both autosomal recessive and dominant inheritance patterns [12,13]. In this scenario, the father may have normal factor VII levels because he carries two normal *F7* genes. The mother, however, may have a mutation - either inherited or de novo - affecting one of her *F7* genes. If this mutation produces a defective factor VII protein with a dominant-negative effect, it could interfere with the function of the normal factor VII produced by her other allele. She could have then passed this mutated gene to all three of her children, explaining why they all exhibit reduced factor VII activity. The varying factor VII levels observed between the mother and her children could be attributed to reduced penetrance. However, this hypothesis requires confirmation through genetic testing. Unfortunately, the family declined further genetic evaluation.

Our suspicion of an autosomal dominant transmission pattern is subject to several limitations, the most significant being the absence of genetic testing in the family. Our analysis is based solely on factor VII activity levels rather than direct genetic confirmation. Factor activity can be influenced by various factors, including consumption, vitamin K levels, and other physiological variables. Moreover, while most coagulation factor deficiencies show a correlation between factor activity and genetic status, this relationship is not always strict. Therefore, the father’s normal factor VII activity does not entirely rule out the possibility that he carries a defective gene but remains asymptomatic. For example, if his normal allele contributes 50% of factor VII activity and a mutated allele contributes 16%, his overall activity could still appear within the normal range. However, if a child inherits this defective allele from the father and a low-activity allele from the mother, they may experience more severe deficiency - potentially explaining the presentation in the two daughters. Conversely, a child inheriting only one defective allele from either parent may have a milder phenotype, as seen in the son.

Nevertheless, the observation of our patient’s family, combined with existing knowledge of factor XI deficiency, presents a unique opportunity to re-evaluate the transmission pattern of factor VII deficiency.

## Conclusions

To our knowledge, this is the first reported case of a pediatric patient with severe factor VII deficiency presenting with menorrhagia leading to life-threatening bleeding. Further testing revealed that only the mother and her three children had decreased factor VII levels, while the father remained unaffected. This intriguing finding provides an opportunity to reconsider the inheritance pattern of factor VII deficiency, which is traditionally classified as autosomal recessive. If the father indeed carries two normal *F7* alleles and only the mother has a defective gene - transmitting it to all three children - this would suggest an autosomal dominant inheritance pattern, at least within this family. However, this hypothesis requires confirmation through genetic testing.
